# Association of Brain‐derived Neurotrophic Factor Polymorphisms With Alcohol Use Disorder: An Updated Meta‐Analysis of Genetic Association Studies

**DOI:** 10.1002/brb3.70359

**Published:** 2025-02-28

**Authors:** Anorut Jenwitheesuk, Noel Pabalan, Pairath Tapanadechopone, Hamdi Jarjanazi, Kittipun Arunphalungsanti, Phuntila Tharabenjasin

**Affiliations:** ^1^ Princess Agrarajakumari College of Nursing Chulabhorn Royal Academy Bangkok Thailand; ^2^ Chulabhorn International College of Medicine Thammasat University Pathum Thani Thailand; ^3^ Department of Psychiatry, Faculty of Medicine Thammasat University Pathum Thani Thailand; ^4^ Environmental Monitoring and Reporting Branch, Ontario Ministry of the Environment, Conservation and Parks Toronto Ontario Canada; ^5^ Department of Physical Therapy, College of Health Sciences Christian University Nakhon Pathom Thailand

**Keywords:** brain‐derived neurotrophic factor, polymorphism, alcohol use disorder, meta‐analysis

## Abstract

**Background:**

Brain‐derived neurotrophic factor (BDNF) has been proposed to play a role in chronic alcohol consumption. However, studies investigating the association of single nucleotide polymorphisms (SNPs) in the *BDNF* gene with alcohol use disorder (AUD), including alcohol dependence, have obtained inconsistent results. This meta‐analysis aims to examine the role of *BDNF* SNPs (rs6265, rs16917204, rs7103411, and rs11030104) in the risk of AUD.

**Materials and Methods:**

A multidatabase search identified 17 articles (20 studies) for inclusion. Pooled odds ratios (ORs) and 95% confidence intervals (CIs) were calculated to estimate associations using standard genetic models (homozygous, recessive, dominant, and codominant). Significant associations were defined as those with a *p*‐value ≤ 0.05 after applying the Bonferroni correction (*p* ^BC^). Subgroup analysis was conducted based on ethnicity (Caucasian and Asian populations). Sources of heterogeneity were investigated through outlier treatment and meta‐regression analysis. Only significant outcomes were further subjected to sensitivity analysis and assessment of publication bias.

**Results:**

This meta‐analysis generated four significant pooled ORs, representing the core outcomes, all of which indicated reduced risks. Overall, the results indicated a significant association between the *BDNF* polymorphism and the risk of AUD in homozygous (OR = 0.72, 95% CIs = 0.60–0.85, *p* ^BC^ = 0.0038) and codominant (OR = 0.84, 95% CIs = 0.78–0.91, *p* ^BC^ = 0.0019) model. In subgroup analysis by ethnicity, homozygous (OR = 0.59, 95% CIs = 0.44–0.78, *p* ^BC^ = 0.0057) and recessive (OR = 0.61, 95% CIs = 0.46–0.81, *p* ^BC^ = 0.0133) models of *BDNF* polymorphisms were significantly associated with a reduced risk of AUD in Caucasians. However, no significant associations were found in Asians. Meta‐regression analysis did not identify any covariates that significantly contributed to the observed heterogeneity. The core significant associations were robust and showed no evidence of publication bias.

**Conclusion:**

The current meta‐analysis suggests that the examined *BDNF* SNPs have a protective effect in the overall analysis (homozygous and codominant) and in the Caucasians subgroup (homozygous and recessive) while the Asians exhibited no effects of *BDNF* SNPs on AUD. *BDNF* polymorphisms might serve as a protective factor against the risk of AUD and could be useful markers in the clinical genetics of AUD.

## Graphical abstract

Graphical abstract of the meta‐analysis conducted to examine the role of BDNF SNPs (rs6265, rs16917204, rs7103411, rs11030104) in the risk of alcohol use disorder.

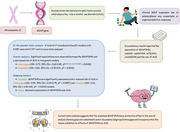

## 1 Introduction

Alcohol use disorder (AUD) is one of the most common serious psychiatric disorders which progresses and worsens over time. This disorder is characterized by recurring cycles of chronic drinking, abstinence, relapse, and behavioral impairments (Su et al. 2011). Approximately 3.3 million deaths worldwide are attributed to harmful alcohol use, including disabilities and medical complications (e.g. liver diseases, gastritis, pancreatitis, and cardiomegaly), making it one of the leading risk factors for the global disease burden (Lim et al. 2012; WHO 2014). AUD is the third leading cause of preventable death and the leading risk factor for premature disability among people aged 15 to 49 years (NIAAA 2024). Previous evidence indicates that both gender‐related factors are interacting with AUD in a complex manner (Slade et al. 2016). Gender gaps in AUD appear to be universal, but significant differences between countries indicate that culturally defined gender roles, and biological sex differences, play a key role in gender‐specific drinking behaviors (White 2020; Wilsnack et al. 2000). In 2016, 54% of males (1.46 billion) and 32% of females (0.88 billion) aged 15 and older worldwide reported consuming alcohol (WHO 2018). Among adolescents and young adults, the gender gap in alcohol consumption has narrowed, primarily due to a greater decline in alcohol use among males compared with females (White 2020)

The psycho‐behavioral manifestations of AUD, including alcohol dependence and abuse, stem from neural pathways involved in regulating motivation, stress, reward, and arousal (Klimkiewicz et al. 2017). These neuronal circuits can undergo changes and adaptations due to chronic alcohol exposure, leading to alcohol‐seeking behavior and alcohol dependence. A number of epidemiological studies have shown that AUD is frequently comorbid with various psychiatric conditions such as schizophrenia, mood and anxiety disorders, and substance abuse (Petrakis et al. 2002; Su et al. 2011; Zai et al. 2018). In addition, AUD is a complex multifactorial disorder with interacting genetic and environmental components (Shin et al. 2010; Zai et al. 2018). The heritability of AUD is estimated to be between 50% and 60%, with modest shared environmental effects (10%) (Pickens et al. 1991; Verhulst et al. 2015).

A growing body of literature suggests brain‐derived neurotrophic factor (BDNF), a member of the ‘neurotrophic’ family of proteins, plays a critical role in susceptibility to substance/drug addiction (Haerian 2013; Su et al. 2011; Raivio et al. 2012). BDNF is primarily distributed in brain regions that regulate mood and behavior, including the hippocampus, cerebellum, hypothalamus, neocortex, and amygdala, with lower levels observed in the amygdala, cerebellum, and cerebral cortex (Hofer et al. 1990; Timmusk et al. 1993). BDNF regulates the proliferation, survival, and differentiation of neurons and modulates and maintains synaptic plasticity in multiple neurotransmitter systems in learning and memory (Gao et al. 2022; Peregud et al. 2023; Shin et al. 2010). Importantly, it also plays a role in the dopaminergic and glutamate systems, which are involved in psychostimulant abuse and dependence (Corominas et al. 2007; Su et al. 2011).

The *BDNF* gene maps to human chromosome 11, band p13, spanning about 70 kb. It consists of 9 functional promoters and 11 exons and is synthesized as a 27 kDa pre‐pro‐BDNF (precursor protein) in the endoplasmic reticulum (Faris et al. 2020). A 32 kDa pro‐BDNF protein is transported into the Golgi apparatus. Proteolytic cleavage of pro‐BDNF by endoproteases or pro‐protein convertases leads to the production of the mature 14 kDa BDNF protein. Single nucleotide polymorphisms (SNPs), the most common type of DNA sequence variation, are known to influence alcohol‐drinking behavior and contribute to the development of AUD (Bach et al. 2015; Hallikainen et al. 1999; Plemenitas et al. 2015). Approximately 40 single nucleotide polymorphisms (SNPs) have been proposed within the *BDNF* gene (Sklar et al. 2002). Among these, the most extensively studied is valine (Val) 66‐to‐methionine (Met) (rs6265/G196A). This SNP involves guanine (G)‐to‐adenine (A) substitution in a highly unstable region at position 196 of the 5′ pro‐region encoding pro‐BDNF, which encodes the precursor of BDNF (proBDNF). While this *BDNF* polymorphism does not affect the function of the mature BDNF protein, the Met allele has been shown to disrupt the intracellular trafficking and packaging of pro‐BDNF in the secretory pathway, thereby reducing the secretion of the mature peptide from neurons (Chen et al. 2004, Chen et al. 2006; Egan et al. 2003; Klimkiewicz et al. 2017). Altered BDNF expression due to polymorphisms may exacerbate or suppress addictive responses.

The impact of Val66Met (rs6265) *BDNF* polymorphism has been widely studied in a variety of drug addictions and psychiatric diseases (Gratacos et al. 2008; Grzywacz et al. 2010; Matsushita et al. 2004; Wojnar et al. 2009). Several studies have identified an association between rs6265 and alcohol abuse, as well as related phenotypes (Benzerouk et al. 2013; Colzato et al. 2011; Shin et al. 2010; Wojnar et al. 2009). The results of human and animal studies consistently show that low levels of mature BDNF are associated with alcohol dependence (Cheah et al. 2014; Matsushita et al. 2004; Wojnar et al. 2009; Zhou et al. 2018). Additionally, low BDNF levels are associated with memory impairment, increased susceptibility to neuropsychiatric disorders such as major depressive disorder and Parkinson's disease as well as substance dependence, including methamphetamine, heroin, cocaine, and nicotine. (Brunoni et al. 2008, Egan et al. 2003; Haerian 2013; Neves‐Pereira et al. 2002; Momose et al. 2002). A study by Elzinga et al. (2011) and Carballedo et al. (2013) demonstrated that carriers of the Met allele who experienced childhood abuse exhibited the lowest serum BDNF levels and reduced hippocampal volumes. Plasma BDNF levels were also found to be lower in suicidal depressed patients compared with nonsuicidal depressed individuals (Kim et al. 2007). In particular, AUD is the most frequently observed comorbidity in individuals with schizophrenia (Drake and Mueser 2002). Previous studies have found that the A allele of rs6265 is associated with comorbid alcohol dependence and risk‐taking behavior after drinking in individuals with schizophrenia (Cheah et al. 2014; Gratacòs et al. 2007; Zai et al. 2018). On the other hand, both positive and negative associations have been reported for Parkinson's disease (Liu et al. 2005), anxiety disorder (Frustaci et al. 2008), depression (Czira et al. 2012), impulsivity (Su et al. 2015), panic disorder (Chen et al. 2017; Xia et al. 2023), and posttraumatic stress disorder (Bountress et al. 2017; Hu et al. 2021).

Notably, observed phenotypic associations in AUD (Uhl et al. 2001) have also been attributed to proximity between the *BDNF* SNPs, referred to as linkage disequilibrium (LD).

LD refers to the correlation between alleles at two or more loci. LD can result in the formation of haplotypes. The presence of SNPs in LD enables an allele of one polymorphic marker to be used as a surrogate for a specific allele of another (Brookes 1999). Multiple groups of SNPs with strong intragroup LD are physically close and inherited together (Takeuchi et al. 2005). The synergistic effect of combining SNPs could enhance the predictive power of the association (Nagel et al. 2014). Regarding *BDNF* polymorphisms, three other SNPs, rs16917204, rs7103411, and rs11030104, have been reported to be in complete LD. In the 3'UTR, a noncoding region, the polymorphism rs16917204 (G11757C) showed no association with alcohol dependence (AD) or AD‐related depression (Su et al. 2011). Instead, it has been associated with bipolar affective disorder and Alzheimer's disease‐related depression (Borroni et al. 2009; Sklar et al. 2002). The only study that specifically investigated intronic SNP rs7103411 (position: chr11:27,656,701) in AUD was Cheah and coworkers (Cheah et al. 2014). Overall, C allele of rs7103411 were associated with comorbid AD and risk‐taking behavior following drinking in the schizophrenia subgroup. Furthermore, the rs6265‐rs7103411 A‐C haplotype was associated with comorbid alcohol dependence and schizophrenia. Additionally, two‐marker BDNF haplotypes encompassing rs11030104 and rs6265 were also reported to be related to AD (Zai et al. 2018).

Although the association between *BDNF* polymorphisms and AUD has been extensively studied, contradictory and inconclusive results have been reported (Cheah et al. 2014; Grzywacz et al. 2010; Nedic et al. 2013; Sery et al. 2011; Shin et al. 2010; Su et al. 2011; Wojnar et al. 2009). To provide a more precise estimation, this study conducted a meta‐analysis to examine the role of *BDNF* SNPs in the risk for AUD, which may offer a better understanding of the genetics of AUD.

## 2 Materials and Methods

### 2.1 Selection of Studies

Four databases (PubMed, Science Direct, Google Scholar, and Mednar) were searched for association studies as of March 28, 2024. The terms used were “brain‐derived neurotrophic factor”, “BDNF”, “polymorphism”, “alcohol use disorder”, “substance dependence”, “alcohol dependence” and “alcoholism” as medical subject headings and text, restricted by English language (Table S1). The additional eligible studies were manually screened and identified from references cited in the retrieved articles. As for duplicate articles, studies with a later date of publication were selected.

The criteria for article inclusion were as follows: (1) human case–control studies examining the association between *BDNF* SNPs and risk of AUD; (2) providing sufficient genotype frequencies of *BDNF* data in the presence and absence of AUD to evaluate AUD risk in terms of ORs and CIs. We excluded the articles if they were (1) review articles, (2) not involving *BDNF*, (3) not involving human subjects, (4) commentaries/editorials, (5) studies not involving AUD, (6) haplotypes, (7) without *BDNF* genotype or unusable data, and (8) non‐English articles.

### 2.2 Data Extraction and SNP Groupings

We examined four *BDNF* SNPs: rs6265, rs16917204, rs7103411, and rs11030104, which are in complete LD (*D*′ and *r* ^2^ = 1.0) based on data from 17 articles (Table 1). Complete LD is determined by *D*′ and *r* ^2^ with values of 1.0 (Borecki 2001; Lewontin 1988). The reason for SNP grouping relies on the theory that SNPs within high LD would have a similar association results.

**TABLE 1:** Characteristics of the included studies in *BDNF* association with AUD.

								Age						
	Gratacòs et al. (2007)	Haerian (2013)	Forero et al. (2015)	First author	Year	Country	Ethnicity	Control	Case	Sex ratio (M:F)	Control	*BDNF* polymorphisms examined	Comorbid psychiatric phenotypes	Clark–Baudouin score
1	Yes	Yes	Yes	Matsushita	2004	Japan	Asian	50.7 ± 17.8	49.9 ± 8.4	Males only	Matched	rs6265	Substance‐related disorder	8
2	^a^	^a^	^a^	Mo	2021	China	Asian	38 (23‐59)^b^	44 (23‐60)^b^	Unspecified	Healthy	rs6265	—	5
3	^a^	^a^	Yes	Shin	2010	Korea	Asian	75.2 ± 5.4	75.2 ± 5.4	Males only	NM	rs6265	—	8
4	^a^	Yes	Yes	Su	2011	China	Asian	44.8 ± 9.2	45.4 ± 10.0	Males only	Healthy	rs6265, rs16917204	Depression	7
5	^a^	^a^	Yes	Tsai	2005	Taiwan	Asian	35.7 ± 15.3	36.1 ± 8.9	Males only	Healthy	rs6265	Extreme violence, psychosis	7
6	^a^	Yes	^a^	Benzerouk	2013	France	Caucasian	34.8 ± 11.2	36.2 ± 11.8	0.31:1	Healthy	rs6265	Executive functions impairment	5
7	^a^	^a^	^a^	Berent	2020	Poland	Caucasian	39.4 ± 12.0	43.4 ± 10.5	3.15:1	Healthy	rs6265	Suicide attempt	8
8	^a^	^a^	Yes	Cheah	2014	Australia	Caucasian	45.0 ± 13.2	40.7 ± 10.3	1.33:1	Healthy	rs6265, rs7103411	Schizophrenia	8
9	^a^	Yes	Yes	Grzywacz	2010	Poland	Caucasian	37.0 ± 8.5	39.0 ± 16.0	6.9:1	Healthy	rs6265	—	7
10	^a^	^a^	^a^	Liu	2005	USA	Caucasian^c^	NA	NA	NA	NM	rs6265	Substance abuse, Parkinson	9
11	^a^	Yes	Yes	Muschler	2011	Germany	Caucasian	38.4 ± 14.8	44.1 ± 8.7	NA	NM	rs6265	—	7
12	^a^	Yes	Yes	Nedic	2013	Croatia	Caucasian	58.5 ± 17.8	50.5 ± 10.7	3.12:1	Healthy	rs6265	Depression, aggression, delirium tremens, suicide attempt, withdrawal syndrome	8
13	^a^	^a^	^a^	Pivac	2022	Croatia	Caucasian	51 (41,58)^b^	49 (43, 56)^b^	2.64:1	Healthy	rs6265	—	8
14	^a^	Yes	Yes	Sery	2011	Czechoslovakia	Caucasian	43.3 ± 8.7	45.4 ± 8.4	Males only	NM	rs6265	Deficient color vision	7
15	^a^	^a^	^a^	Wojnar	2009	USA	Caucasian^c^	NA	44.2 ± 10.1	3.2:1	Abstinent	rs6265	—	9
16	^a^	^a^	^a^	Zai	2018	Canada	Caucasian^c^	NA	37.9 ± 10.7	1.95:1	Unspecified	rs6265, rs11030104	Schizophrenia	7
17	^a^	^a^	^a^	Zhang	2006	USA	Caucasian^c^	37.5 ± 19.7	39.8 ± 9.4	1.51:1	Healthy	rs6265	Alzheimer's disease, affective disorders, posttraumatic stress disorder, Schizophrenia, Substance dependence	9

*Note*: All articles examined rs6265 as a common single nucleotide polymorphism.

Abbreviations: AUD, alcohol use disorder; *BDNF*, brain‐derived neurotrophic factor; F, female.; M, male; NA, nonapplicable; NM, no mention.

^a^ The article did not include in the previous meta‐analysis.

^b^ Median (range).

^c^ All European American.

A. J. and K. A. independently performed data extraction and N. P. validated and arrived at a consensus. The following information from each publication was determined: whether the article was included in a previous meta‐analysis, first author's name, a year of publication, country of articles published, ethnicity, age of the subjects (control and case), sex ratio, control status, *BDNF* polymorphisms examined, comorbid psychiatric phenotype, and quality assessment scale for the included studies by the Clark–Baudouin score (Table 1)

### 2.3 Data Synthesis

The normality of data distribution was evaluated by the Shapiro–Wilks test using SPSS 20.0 (IBM Corp., Armonk, NY, USA). Descriptive statistics and inferential expressions of mean ± standard deviation (SD) as well as parametric tests were applied to data showing normal distributions (Gaussian distribution at *p* > 0.05). Otherwise, nonparametric tests and the median with interquartile range were used.

Statistical power analyses were computed using the G* Power program (Faul et al. 2007), assuming an OR of 1.5 at a genotypic risk level of *α* = 0.05 (two‐sided). High statistical power of data was considered at ≥75%. Assessment of HWE from genotype frequencies was tested by using the application in https://gene‐calc.pl/hardy‐weinberg‐page. Departures of genotypic frequencies from the HWE in control subjects were determined with Pearson's goodness‐of‐fit *χ* ^2^‐square test (*p* < 0.05; Table 2). Laplace correction was applied when genotype frequency values were zero (Berthold et al. 2010). By this method, all values of the data set were added to a pseudocount of one before generating the forest plots (Table 2).

**TABLE 2:** Quantitative features of the included *BDNF* gene polymorphism studies that examined associations with AUD.

								Case			Control				
	First author	Ethnicity	Polymorphism	Case 4095	Control 4727	Total 8822	Power^a^ (%)	wt–wt	var–wt	var–var	wt–wt	var–wt	var–var	MAF	*p*‐HWE
1	Matsushita	Asian	rs6265	377	336	713	**75.9**	141	176	60	106	162	68	0.443	0.67
2	Shin	Asian	rs6265	68	232	300	30.4	8	41	19	61	127	44	0.463	0.13
3	Su	Asian	rs6265	548	312	860	**80.4**	175	250	123	101	148	63	0.439	0.51
4	Su	Asian	rs16917204	^b^	^b^	^b^	^b^	252	222	74	137	128	47	0.356	0.06
5	Tsai	Asian	rs6265	110	149	259	35.4	25	63	22	29	85	35	0.520	0.08
6	Mo	Asian	rs6265	59	37	96	65.5	17	33	9	8	20	9	0.513	0.62
7	Berent L	Caucasian	rs6265	176	127	303	40.2	117	58	1	94	30	3	0.142	0.74
8	Benzerouk	Caucasian	rs6265	46	82	128	19.1	30	14	2	47	30	5	0.244	0.94
9	Cheah	Caucasian	rs6265	42	98	140	19.1	22	18	2	64	33	1	0.179	0.14
10	Cheah	Caucasian	rs7103411	41	100	141	18.8	20	19	2	66	32	2	0.172	0.18
11	Grzywacz L	Caucasian	rs6265	138	153	291	39.7	91	46	1	107	42	4	0.163	0.96
12	Liu	Caucasian	rs6265	322	322	644	71.7	243	73	6	206	103	13	0.200	0.98
13	Muschler	Caucasian	rs6265	239	99	338	38.6	151	83	5	68	28	3	0.172	0.87
14	Nedic	Caucasian	rs6265	675	915	1590	**97.6**	458	197	20	609	274	31	0.184	0.98
15	Pivac	Caucasian	rs6265	650	918	1568	**97.4**	440	190	20	600	263	55	0.203	**0.0005**
16	Sery	Caucasian	rs6265	167	289	456	53.7	109	55	3	195	89	5	0.171	0.15
17	Wojnar	Caucasian	rs6265	59	60	119	19.1	39	17	3	27	25	8	0.342	0.57
18	Zai L	Caucasian	rs6265	25	124	149	14.8	22	5	1	69	48	10	0.258	0.82
19	Zai L	Caucasian	rs11030104	26	124	150	15.1	21	7	1	65	51	11	0.282	0.96
20	Zhang	Caucasian	rs6265	327	250	577	66.2	220	100	7	166	74	10	0.188	0.63

*Note*: HWE, Hardy‐Weinberg Equilibrium (*p*‐values where ≤.05 is significant). Values in bold indicate statistically powered studies (> 75%). Single nucleotide polymorphisms in complete linkage disequilibrium: rs6265, rs16917204, rs7103411, and rs11030104.

Abbreviations: AUD, alcohol use disorder; *BDNF*, brain‐derived neurotrophic factor; L, Laplace correction; maf, minor allele frequency; var, variant; wt, wild type.

^a^ *α* = .05; OR  = 1.5.

^b^ Duplicate data.

### 2.4 Methodological Quality of the Studies

The methodological quality of the included studies was evaluated by the Clark–Baudouin score (Clark and Baudouin 2006). The criteria of the assessment were based on comparative sample sizes between cases and controls, *p*‐values, statistical power, use of primers and detailing of genotyping methods, correction for multiplicity, and the HWE. The scores of < 5, 5–6, and ≥7 indicated low, moderate, and high quality, respectively.

### 2.5 Meta‐analysis

The articles included in our study consist of genotypes of *BDNF* SNPs with different rs numbers. Therefore, we selected the generic wild‐type (wt) and variant (var) notations in this meta‐analysis. The results were presented by using four genetic models including (1) homozygous: (var–var and wt–wt) genotypes compared with wt–wt; (2) recessive: var–var versus var–wt + wt–wt; (3) dominant: var–var + var–wt versus wt–wt; and (4) codominant (multiplicative): var versus wt. Comparing effects on the same baseline, we used raw data for genotype frequencies to calculate pooled ORs and corresponding 95% Cis. To reduce the occurrence of false positives, Bonferroni correction was applied to all *p*‐values from multiple associative comparisons. The significant association was considered when the Bonferroni corrected *p*‐value (*p* ^BC^) ≤0.05.

We subgrouped the individual meta‐analyses based on ethnicity, which are Caucasians and Asians. The variation across studies resulting from heterogeneity was evaluated by the χ^2^‐based *Q* test (Higgins and Thompson 2002; Higgins et al. 2003) with a threshold of significance set at *p* ^het^ < 0.10. The presence of heterogeneity warranted the use of the random‐effects model (DerSimonian and Laird 1986), otherwise, the fixed‐effects model was used (Mantel and Haenszel 1959). The Galbraith plot analysis was used to detect the outlier studies which are sources of heterogeneity (Galbraith 1988). The outcomes of our meta‐analysis were dichotomized into preoutlier treatment and postoutlier treatment (removed the source of heterogenous study) comparisons

To identify sources of heterogeneity across studies, meta‐regressions with specific covariates of personal factors (for example, age, ethnicity, and sample size) were performed using the Open Meta‐Analyst software (Wallace et al. 2009). A significant *p*‐value with Bonferroni correction (*p* ^BC^) was set at ≤0.05.

Sensitivity analysis, which involves omitting one study at a time and recalculating the pooled OR, was used to test for the robustness of the summary effects. Only significant outcomes (*p* ^BC^ ≤ 0.05) with >10 studies were further analyzed for publication bias (Ioannidis and Trikalinos 2007). Publication bias was assessed using WINPEPI (Abramson 2004). Study‐specific ORs were used as operating data for the publication bias tests, with the selection of the test depending on the data distribution. For normally distributed data, Egger's test (Egger et al. 1997) was applied, while the Begg–Mazumdar test (Begg and Mazumdar 1994) was used for nonnormally distributed data.

Data for the meta‐analysis were analyzed using Review Manager 5.4 (Cochrane Collaboration, Oxford, England), SIGMASTAT 2.03, and SIGMAPLOT 11.0 (Systat Software, San Jose, CA).

## 3 Results

### 3.1 Search Results

Following the Preferred Reporting Items for Systematic Reviews and Meta‐Analyses guidelines (Table S3; Moher et al. 2009), Figure 1 outlines the study selection process in a flowchart. The initial search yielded 6645 citations based on our search strategies. After screening titles and abstracts and removing duplicates, 92 articles were selected for further evaluation. These articles were then screened to exclude reviews, commentaries, editorials, and studies that did not involve *BDNF* polymorphism, human subjects, or AUD. A total of 23 full‐text articles were further assessed for eligibility according to the inclusion criteria. Ultimately, 17 articles were included in the meta‐analysis (Benzerouk et al. 2013; Berent et al. 2020; Cheah et al. 2014; Grzywacz et al. 2010; Liu et al. 2005; Matsushita et al. 2004; Mo et al. 2021; Muschler et al. 2011; Nedic et al. 2013; Pivac et al. 2022; Sery et al. 2011; Shin et al. 2010; Su et al. 2011; Tsai et al. 2005; Wojnar et al. 2009; Zai et al. 2018; Zhang et al. 2006).

**FIGURE 1:**
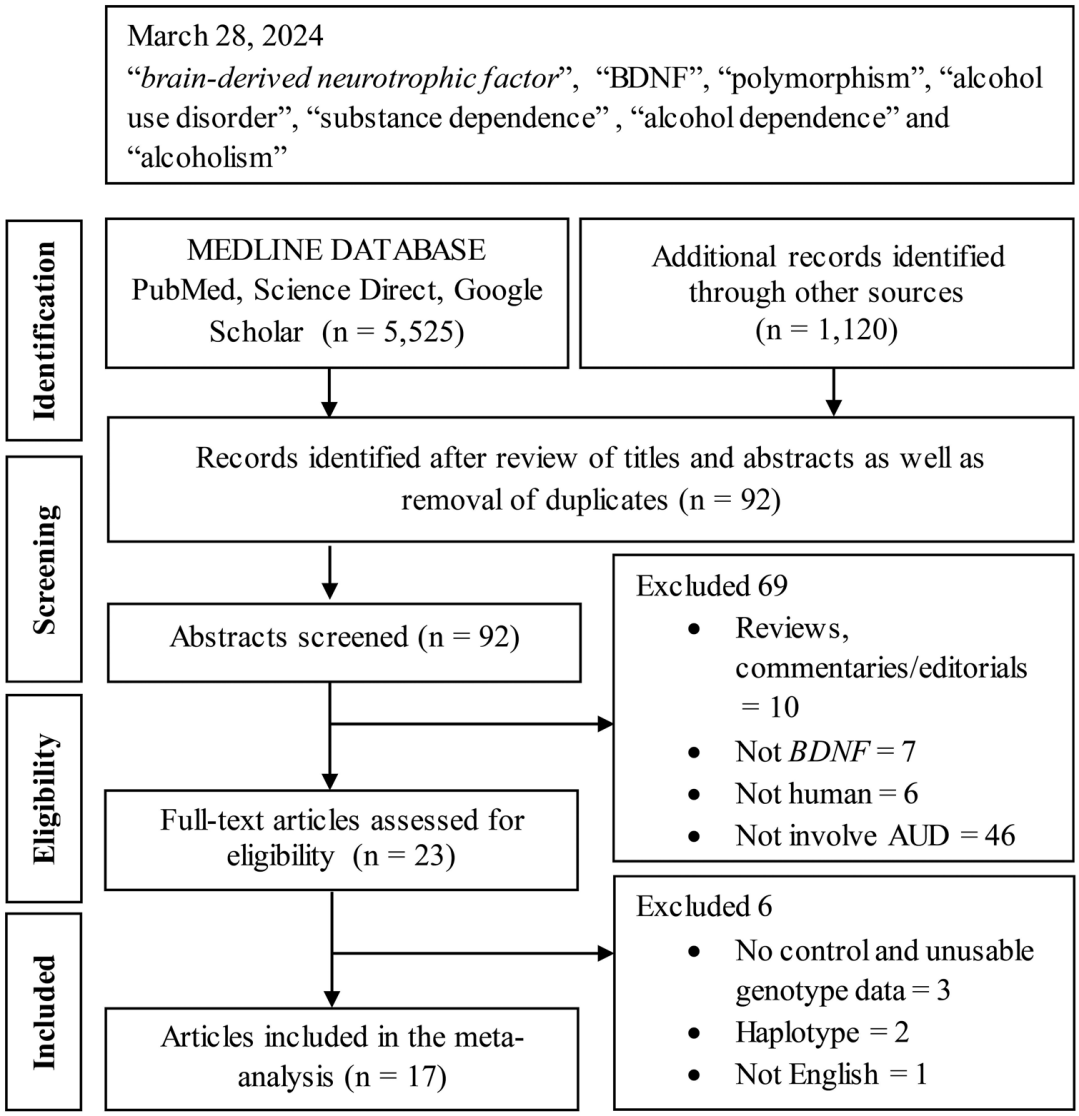
Summary flow chart of literature search. *BDNF*, brain‐derived neurotrophic factor; *n*, number of articles; AUD, alcohol use disorder.

All of the included articles examined rs6265 as a common SNP (Table 1). Three articles (Cheah et al. 2014; Su et al. 2011; Zai et al. 2018) dealt with additional *BDNF* polymorphisms in complete LD with rs6265. The numbers of cases and controls were 4095 and 4727, respectively (Table 2). Of the 17 articles (20 studies), 6 and 14 studies were Asian and Caucasian, respectively. The age of the subjects (mean of the mean) indicated a middle‐aged demographic profile (mean 41.2 ± SD 4.04). For the most part, male subjects outnumbered females by a factor as high as 6.9 males per female. In fact, five studies used males only (Matsushita et al. 2004; Sery et al. 2011; Shin et al. 2010; Su et al. 2011; Tsai et al. 2005), and 58.8% of articles (10/17) had healthy controls (Table 1). The comorbid psychiatric phenotypes of AUD in all included studies consist of substance‐related disorders, depression, extreme violence, psychosis, executive function impairments, suicide attempts, schizophrenia, Parkinson's disease, aggression, delirium tremens, withdrawal syndrome, Alzheimer's disease, affective disorders, and posttraumatic stress disorder. The Clark–Baudouin average scores (7.47) indicated that the methodological quality of the component studies was high (Table 1). Four articles (Matsushita et al. 2004; Nedic et al. 2013; Pivac et al. 2022; Su et al. 2011) with five studies were statistically powered (≥75%) (Table 2). The frequency difference of the minor allele was significant (*t* = −7.79; *p* < 0.0001) between Caucasian (mean 0.21 ± SD 0.06) and Asian (mean 0.44 ± SD 0.06) ethnicities (Table 2). One (Pivac et al. 2022) of 20 studies was not HWE‐compliant (Table 2).

### 3.2 Meta‐analysis

Table 3 summarizes the meta‐analysis outcomes by order of genetic model. Of 20 studies overall, 14 studies from Caucasian populations and 6 from Asians were used for pooling. After correcting the data for multiple comparisons using the Bonferroni method, four results remained significant (*p* ^BC^ ≤ 0.05). Pooled odds ratios (ORs) less than 1.0 indicate a reduced risk, while ORs greater than 1.0 signify an increased risk.

**TABLE 3:** Analysis of overall and subgroup associations of the *BDNF* gene polymorphisms with AUD.

		Test of association				Test of heterogeneity				Test of association				Test of heterogeneity		
	*n*	OR	95% CI	*p* ^a^	*p^BC^*	*p* ^het^	*I* ^2^ (%)	Analysis model	*n*	OR	95% CI	*p* ^a^	*p^BC^*	*p* ^het^	*I* ^2^ (%)	Analysis model
			Preoutlier treatment								Postoutlier treatment					
Overall																
Homozygous	20	0.76	0.58–0.98	0.04	0.76	0.04	39	Random	17	0.72	0.60–0.85	0.0002	0.0038^a^	0.50	0	Fixed
Recessive	20	0.82	0.71–0.96	0.01	0.19	0.20	21	Fixed	—	—–	———‐	——	——	—	—	—
Dominant	20	0.94	0.80–1.09	0.43	> 1	0.0006	58	Random	15	0.91	0.81–1.00	0.04	0.76	0.11	33	Fixed
Codominant	20	0.90	0.79–1.03	0.13	> 1	0.0001	66	Random	13	0.84	0.78–0.91	<0.0001	0.0019^a^	0.13	32	Fixed
Caucasian																
Homozygous	14	0.59	0.44–0.78	0.0003	0.0057^a^	0.41	4	Fixed	—	—–	———‐	——	——	—	—	—
Recessive	14	0.61	0.46–0.81	0.0007	0.0133^a^	0.62	0	Fixed	—	—–	———‐	——	——	—	—	—
Dominant	14	0.92	0.75–1.13	0.44	>1	0.0005	64	Random	10	1.02	0.91–1.15	0.70	>1	0.25	21	Fixed
Codominant	14	0.88	0.74–1.06	0.20	>1	0.0002	67	Random	9	1.03	0.92–1.16	0.63	>1	0.28	18	Fixed
Asian																
Homozygous	6	0.94	0.64–1.37	0.74	>1	0.03	60	Random	5	0.85	0.68–1.06	0.14	>1	0.37	7	Fixed
Recessive	6	0.95	0.78–1.14	0.57	>1	0.21	30	Fixed	—	—–	———‐	——	——	—	—	—
Dominant	6	0.94	0.80–1.10	0.44	>1	0.10	45	Fixed	—	—–	———‐	——	——	—	—	—
Codominant	6	0.93	0.75–1.14	0.47	>1	0.0007	69	Random	4	0.91	0.80–1.04	0.17	>1	0.26	25	Fixed

Abbreviations: AUD, alcohol use disorder; *BDNF*, *brain‐derived neurotrophic factor*; CI, confidence interval; *I* ^2^, measure of variability.; *n*, number of studies; OR, odds ratio; *p* ^a^, *p*‐value for association; *p* ^het^, *p*‐value for heterogeneity.

^a^ Values in bold indicate a significant association when the Bonferroni‐corrected *p*‐value (*p* ^BC^) is ≤0.05.

In the overall analysis, two significant outcomes were observed: the homozygous model (OR = 0.72, 95% CIs = 0.60–0.85, *p* ^BC^ = 0.0038) and the codominant model (OR = 0.84, 95% CIs = 0.78–0.91, *p* ^BC^ = 0.0019). Both of these indicated a reduced risk and were derived from postoutlier treatment (*p* ^het^ = 0.13–0.50, *I* ^2^ = 0–32%, fixed effect).

Subsequently, a subgroup analysis based on ethnic differences revealed a decreased risk in Caucasian populations, while no significant associations were found in the Asian subgroup (ORs = 0.85–0.95, 95% CIs = 0.64–1.37, *p* ^BC^ > 1, *p* ^het^ = 0.0007–0.37, *I* ^2^ = 7–69%). A decreased risk of *BDNF* SNPs in Caucasians was observed only in preoutlier treatment, with a fixed effect in the homozygous model (OR = 0.59, 95% CIs = 0.44–0.78, *p* ^BC^ = 0.0057, *I* ^2^ = 4%) and the recessive model (OR = 0.61, 95% CIs = 0.46–0.81, *p* ^BC^ = 0.0133, *I* ^2^ = 0%).

Table 4 presents the results of the publication bias assessment for four significant outcomes (*p* ^BC^ ≤ 0.05) and shows no evidence of publication bias in any of the significant pooled ORs. The sensitivity of the significant findings was robust for all, except for the overall analysis in the codominant model.

**TABLE 4:** Assessment of sensitivity analysis and publication bias.

Comparison (*n*)	Genetic model	Status	Sensitivity outcome	Shapiro–Wilks *p*‐value	Normal distribution	Kendalls tau	*p*‐value	Evidence of publication bias
Overall								
17	Homozygous	Postoutlier	Robust	<0.001	No	−0.32	0.010	No
13	Codominant	Postoutlier	Nonrobust	<0.004	No	−0.37	0.010	No
Caucasian								
14	Homozygous	Preoutlier	Robust	<0.001	No	0.27	0.243	No
14	Recessive	Preoutlier	Robust	<0.001	No	0.08	0.714	No

*Note*: With nonnormal distribution of OR, all comparisons underwent the Begg and Mazumdar test for publication bias test.

Abbreviation: *n*, number of studies.

The results of the meta‐regression showed that none of the covariates (year, age, methodological quality, ethnicity, sex ratio, and sample size; *p* ^BC^ ≥ 0.05) contributed to the sources of variability or heterogeneity or among the study included in our study (Table S2).

### 3.3 Mechanism of Outlier Treatment

**FIGURE 2:**
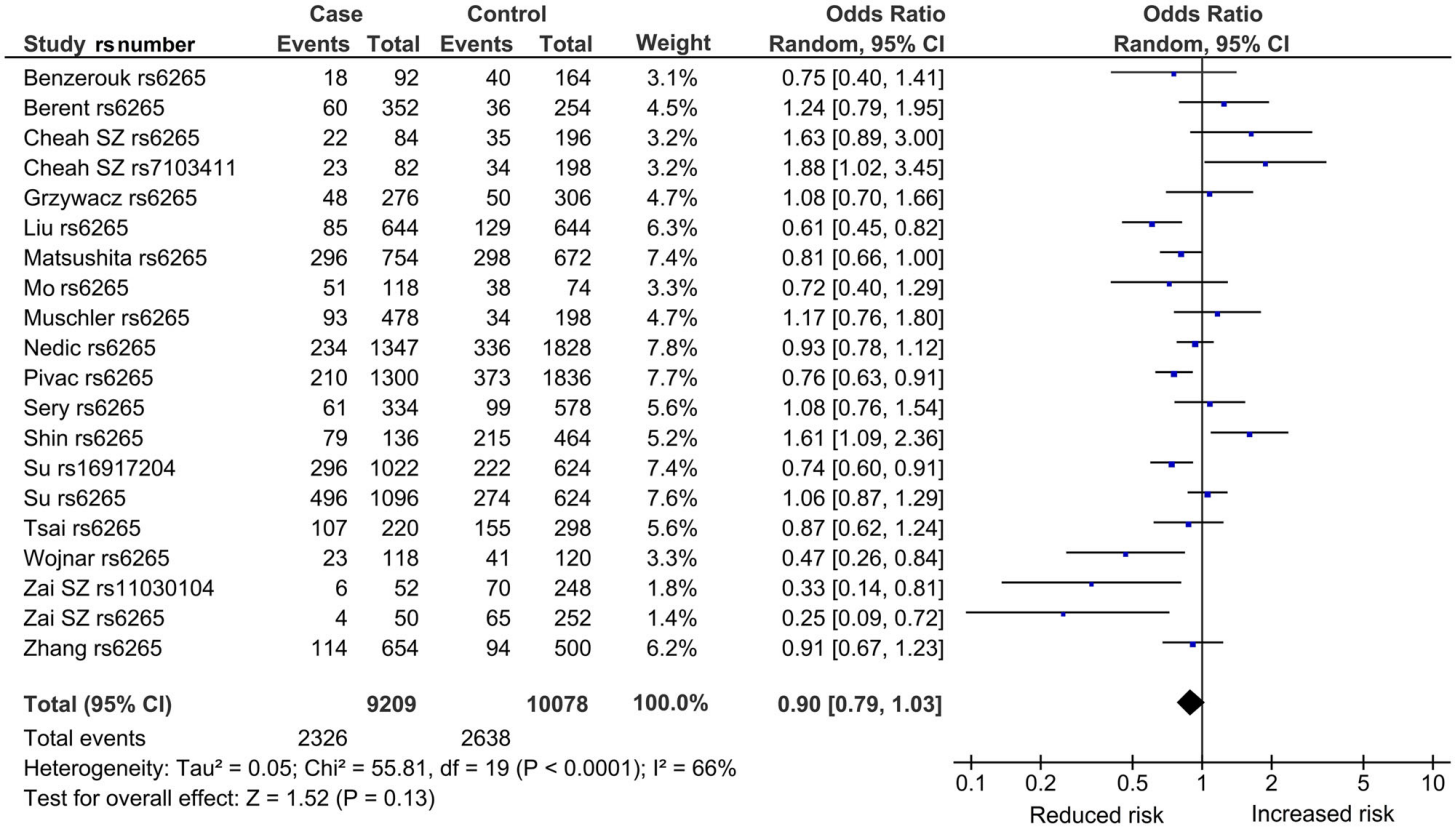
Preoutlier forest plot in the codominant model of *BDNF* of the overall analysis. CI, confidence interval; *BDNF*, brain‐derived neurotrophic factor; df, degree of freedom; *I* ^2^, measure of variability.

**FIGURE 3:**
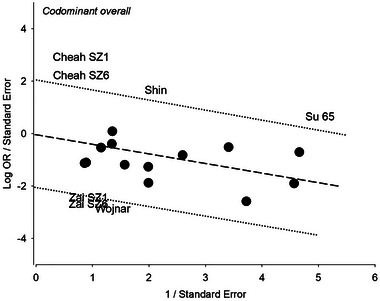
Galbraith plot of the overall analysis in the codominant model showing the outlying studies found below the −2 confidence limit.

**FIGURE 4:**
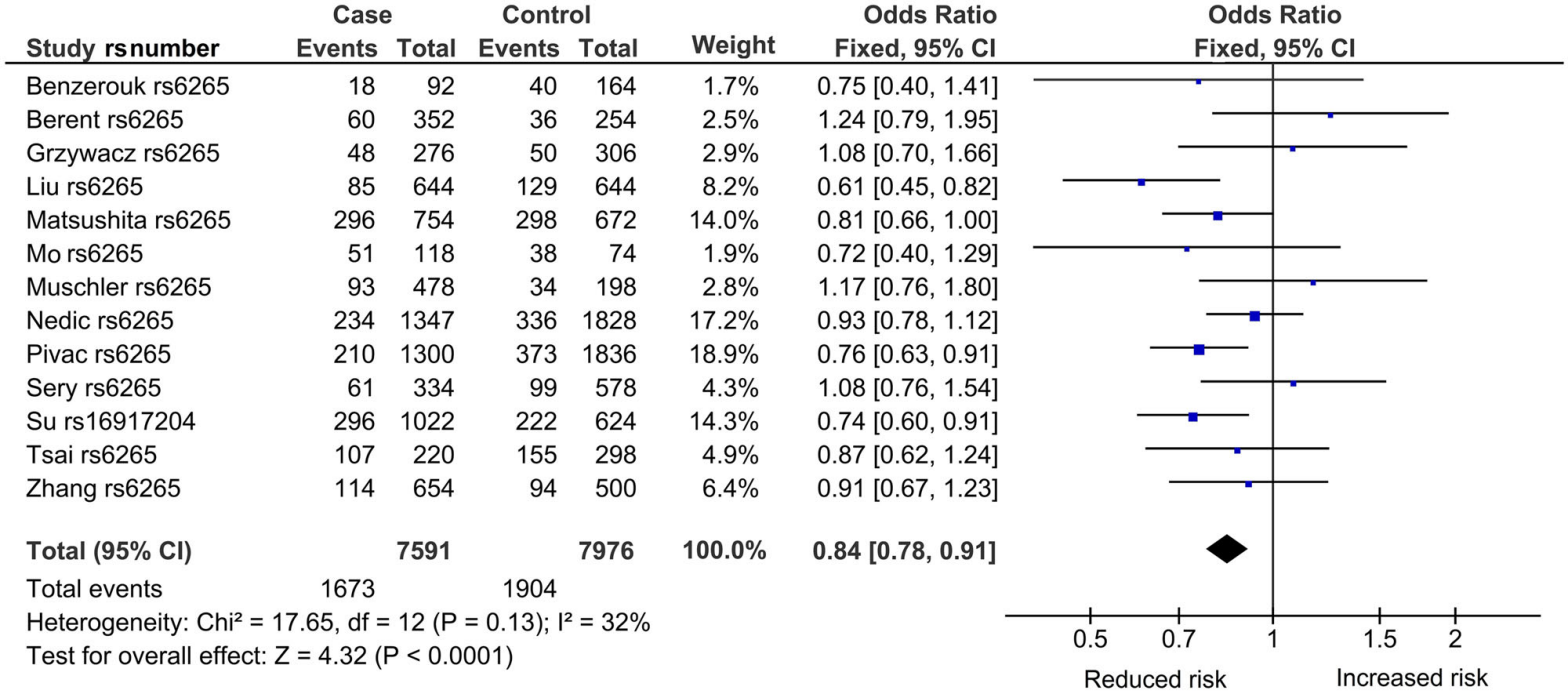
Postoutlier forest plot in the codominant model of *BDNF* of the overall analysis. CI, confidence interval; *BDNF*, brain‐derived neurotrophic factor; df, degree of freedom; *I* ^2^: measure of variability.

The mechanism of outlier treatment is presented for the codominant model in the overall analysis (Figures 2, 3, 4). Figure 2 shows the preoutlier treatment forest plot, with a pooled OR (OR = 0.90, 95% CI = 0.79–1.03), which was nonsignificant (*p* ^a^ = 0.13, *p* ^BC^ > 1) and heterogeneous (*p* ^het^ < 0.00001, *I* ^2^ = 66%). The Galbraith plot identified five outliers (Cheah et al. 2014; Shin et al. 2010; Su et al. 2011; Wojnar et al. 2009; Zai et al. 2018) from seven studies, found above and below the −2 and +2 confidence limits (Figure 3). In Figure 4, the postoutlier treatment outcome (with outlier studies omitted) showed reduced heterogeneity (*p* ^het^ = 0.13, *I* ^2^ = 32%) and gained significance (OR = 0.84, 95% CI = 0.78–0.91, *p* ^a^ = 0.0001, *p* ^BC^ = 0.0019). This operation is numerically summarized in Table 3.

## 4 Discussion

### 4.1 Summary of Findings

This updated meta‐analysis of the pooled data demonstrated a significant association between *BDNF* polymorphisms and AUD in the overall analysis and Caucasian populations while showing no association in Asian populations. A reduced risk of AUD associated with *BDNF* polymorphisms was observed in four significant outcomes: the homozygous and codominant models in the overall analysis before outlier treatment, and the homozygous and recessive models in the Caucasian subgroup after outlier treatment. The core significant outcomes were robust and exhibited no evidence of publication bias. Outlier treatment attempts to resolve heterogeneity issues that are inherent in meta‐analysis, while Bonferroni correction reduces the possibility of false‐positive. Together, these methods strengthen the evidence presented and enhance the transparency of our findings.

### 4.2 *BDNF* and AUD

A neuromodulator BDNF (mature BDNF; mBDNF) provides trophic support and plays a crucial role in the growth, survival, differentiation, and repair of dopaminergic, GABAergic, cholinergic, and serotonergic neurons, which are involved in the rewarding and reinforcing effects of drugs of abuse (Autry and Monteggia 2012; Binder and Scharfman 2004; Kenny et al. 2000; Russo et al. 2009; Vasconcelos et al. 2015). In vitro and in vivo studies strongly suggest that BDNF is implicated in both alcohol preference and aggressive behavior (Raivio et al. 2012; Sadri‐Vakili et al. 2010).

BDNF is initially synthesized as a precursor molecule, proBDNF, which is enzymatically cleaved by matrix metalloproteinase‐7 (MMP‐7) and tissue plasminogen activator (tPA) into mature BDNF (mBDNF) and the BDNF predomain (Le and Friedman 2012; Pang et al. 2004). The conversion of pro‐BDNF to mBDNF is a crucial step in the negative regulation of BDNF actions in the brain. Previous studies suggest that the precursor and mature forms of BDNF interact with distinct receptor/signaling systems, inducing opposing biological effects on neuronal survival, differentiation, and plasticity (Yang et al. 2014; Zhou et al. 2018). Mature BDNF, through its high‐affinity receptor tropomyosin receptor kinase B (TrkB), plays a pivotal role in mediating neuronal survival and growth, while its precursor, proBDNF, can induce neuronal apoptosis through the JNK pathway by binding to the low‐affinity p75 neurotrophin receptor (p75NTR) and sortilin (Teng et al. 2005; Zhou et al. 2018).

Both proBDNF and mBDNF play roles in the pathophysiology of AUD. In alcohol‐dependent (AD) patients, the balance between the proBDNF/p75NTR and mBDNF/TrkB signaling pathways was dysregulated. The results indicated that the expression of the proBDNF/p75NTR pathway was significantly enhanced, whereas the mBDNF/TrkB pathway was suppressed, suggesting that the balance between neurotrophic and neurodegenerative processes was disrupted. Previous studies reported the plasma ratio of proBDNF to mBDNF was significantly higher in the alcohol‐dependence (AD) group compared with the control group (Mo et al. 2021; Zhou et al. 2018). A similar result was also observed in an animal study, which showed increased p75NTR expression in the hippocampus of a dog model of chronic alcoholism, whereas changes in BDNF and TrkB were opposite to those of p75NTR (Xu et al. 2015).

Regarding the association between the *BDNF* Val66Met (rs6265) SNP and neuropsychiatric disorders, This polymorphism has been linked to the magnitude of mBDNF release. The *BDNF* Met allele has been associated with a decreased ability of pro‐BDNF to be packaged in the Golgi apparatus into secretory vesicles, leading to a reduction in the secretion of mBDNF protein into the synapse (Egan et al. 2003; Faris et al. 2020; Nguyen et al. 2023). Furthermore, the polymorphism also affects the downstream signaling pathway of BDNF (Nguyen et al. 2023). In their study, Mo et al. (2021) found that, in AD patients, the plasma level of proBDNF was slightly higher in those with the Met/Met (AA) genotype compared with those with the Val/Val (GG) and Val/Met (AG) genotypes, while the level of mBDNF was slightly lower. Moreover, the plasma level of proBDNF showed a positive correlation with both the average daily alcohol consumption and the duration of alcohol use, while mBDNF showed a negative correlation. In an older Korean population, Shin et al. (2010) reported that men with AUD had higher Met allele and lower Val allele frequencies compared with the control group. However, some studies from other populations reported lower frequencies of the Met allele than the Val allele in AUD patients or found no association with AUD (Grzywacz et al. 2010; Liu et al. 2005; Matsushita et al. 2004; Muschler et al. 2011; Sery et al. 2011; Tsai et al. 2005; Zhang et al. 2006). Of interest, one study found that the Val/Val genotype was also associated with a higher risk and earlier occurrence of relapse among patients treated for AD (Wojnar et al. 2009). As mentioned earlier, AUD is often comorbid with other psychiatric disorders. Previous studies have also reported an association between the AA genotype and A allele frequencies in individuals with AUD, particularly those with co‐occurring schizophrenia, impaired executive functions, violent tendencies, or depression (Benzerouk et al. 2013; Cheah et al. 2014; Matsushita et al. 2004; Su et al. 2011).

Regarding the other three *BDNF* SNPs; rs16917204 (G > A), rs7103411 (C > T), and rs11030104 (C > T), no previous studies have examined the association between these variant alleles and plasma or brain levels of proBDNF and mBDNF. Although all three SNPs are located in the intronic region and do not directly alter the protein‐coding sequence, variations in introns can influence gene regulation or splicing, potentially affecting BDNF expression. One study of haplotype analysis revealed that rs6265‐rs7103411 A/C haplotype is associated with comorbid AD in schizophrenia patients (Cheah et al. 2014). It could be speculated that the plasma level of proBDNF may be higher in individuals with the CC genotype of rs7103411 compared with those with the CT and TT genotypes. Further investigation into the influence of SNPs rs16917204, rs7103411, and rs11030104 on plasma or brain levels of proBDNF and mBDNF in AUD patients without any comorbidities is needed to verify this hypothesis.

This meta‐analysis includes data from 20 case‐control AUD studies. Our results studies are inconsistent with the two previous meta‐analyses by Haerian and colleagues (7 studies, 2013) and Forero and co‐workers (9 studies, Forero et al. 2015), but the study is in agreement with those of Gratacòs and associates (2 studies, Gratacòs et al. 2007). In particular, Gratacòs's study showed a protective effect of the Met allele of *BDNF* for substance‐related disorders, whereas no associations were found in the studies by Haerian and Forero. However, only Forero and co‐workers conducted a meta‐analysis specifically focused on AUD patients. The discrepant results may be due to differences in the number of included studies, which could impact the statistical power of the analysis. Patient‐related phenotypes and quality control of genotypes in the included studies can cause bias and produce false positives. Additionally, previous meta‐analyses have also found that the variant allele of *BDNF* SNPs is associated with an increased risk of disease across clinically diagnosed neuropsychiatric disorders, such as anxiety disorders, methamphetamine addiction, panic disorder, and posttraumatic stress disorder (Bountress et al. 2017; Frustaci et al. 2008; He et al. 2020; Xia et al. 2023).

Ethnicity is another important factor in susceptibility to AUD. Our data found an association between *BDNF* polymorphism and AUD in Caucasians, but not in Asians. This finding is consistent with several studies (Gratacos et al. 2008; Nedic et al. 2013; Petryshen et al. 2010; Pivac et al. 2009), but it is inconsistent with a previous meta‐analysis (Haerian 2013). Our study showed that *BDNF* SNPs have a protective effect in the homozygous and recessive models in the Caucasian subgroup. Ethnic differences in the frequency of the *BDNF* Val66Met alleles and genotypes were demonstrated in large groups of healthy Caucasian and Asian participants. Pivac et al. (2009) reported that the Val/Val genotype was most frequent in Caucasian participants, while the Met/Val genotype was most frequent in Asian participants. The majority of Caucasian individuals were carriers of the Val allele. Moreover, the distribution of the Met and Val alleles was almost equal in the Asian population. In addition, the discrepancies in outcomes between these two populations may result from differences in the genetic background of enzymes involved in alcohol metabolism, such as alcohol dehydrogenase 1B. The fast alcohol‐metabolizing ADH1B T variant causes rapid acetaldehyde accumulation, thereby inhibiting alcohol consumption. One previous study reported that T allele carriers are more prevalent among Asians than Caucasians (Lin et al. 2021). The different distribution of the allele among populations is likely a result of migration, genetic drift, and selection processes. Furthermore, genetic factors may interact with environmental factors such as regional climate, culture, and pathogens, leading to diverse adaptations among populations and individuals (Sabeti et al. 2006; Tishkoff and Verrelli 2003)

The complexity of AUD involves interactions between genetic and nongenetic factors, highlighting the likelihood of environmental involvement. Gene‐gene and gene‐environment interactions have been reported to play roles in the associations of other SNPs with AUD (Katsarou et al. 2017). In this study, only one of the 17 articles mentioned gene–environment interactions (Nedic et al. 2013).

### 4.3 Novelties of the Present Meta‐analysis

The difference between our meta‐analysis with the previous three meta‐analyses (Table 5) include (1) the number of included articles: 17 versus nine (Forero et al. 2015), seven (Haerian 2013), and two (Gratacos et al. 2008); (2) number of *BDNF* SNPs examined: four SNPs in complete LD (rs6265, rs16917204, rs7103411, and rs11030104) versus one (rs6265) in previous three meta‐analyses; (3) significant associations: significantly reduced risk versus no significance in all genetic models; (4) with outlier treatment versus none; (5) Bonferroni correction: applied versus none except Haerian (2013); (6) meta‐regression analysis: applied versus none. Of note, the two meta‐analyses (Gratacos et al. 2008; Haerian 2013) examined substance abuse/drug addiction, which is broad terminology (Wang et al. 2012). Importantly, our study delineated no association (Forero et al. 2015; Gratacos et al. 2008; Haerian 2013) of *BDNF* SNPs and AUD in Asian, which was not reported in previous meta‐analysis and genome‐wide association study (Uhl et al. 2001).

**TABLE 5:** Comparisons between meta‐analyses involving associations between the *BDNF* gene polymorphisms and AUD.

	This study	Forero et al. (2015)	Haerian (2013)	Gratacos et al. (2008)
Year	2024	2015	2013	2008
Country	Thailand	USA/Korea	Malaysia	Spain
number of articles/studies	17/20	9	7	2
Outcomes	Alcohol dependence	Alcohol dependence	Drug Addiction	Substance use disorders
*BDNF* SNP(s)	rs6265	rs6265	rs6265	rs6265
	rs16917204			
	rs7103411			
	rs11030104			
Genetic model	Homozygous	Recessive	Recessive	Genetic
	Recessive	Dominant	Dominant	Free
	Dominant	Codominant	Codominant	Model
	Codominant			
Databases search	PubMed	PubMed	PubMed	PubMed
	Google Scholar		Embase	
	Science Direct		Cochrane	
	Mednar			
Subgroup analysis	Ethnic	Ethnic	Ethnicity, drug type	——–
Methodological quality	Clark–Baudouin	None	Diagnostic and statistical manual of mental disorders	None
Hardy‐Weinberg equilibrium	Yes	Yes	Yes	Yes

Abbreviations: AUD, alcohol use disorder; *BDNF*, brain‐derived neurotrophic factor; SNPs, single nucleotide polymorphisms.

### 4.4 Strengths and Limitations

Interpreting our findings requires considering both their strengths and limitations. Limitations include: (1) psychiatric disorders that are comorbid with AUD may have been confounding factors affecting our results. Three studies examined AUD in schizophrenia patients (Cheah et al. 2014; Zai et al. 2018; Zhang et al. 2006). One study examined AUD in extremely violent males (Tsai et al. 2005). Some studies investigated AUD in depressive‐suicidal patients (Berent et al. 2020; Nedic et al. 2013; Su et al. 2011), and (2) one of the 20 included studies investigated the proBDNF levels corresponding to gene polymorphisms.

On the other hand, strengths of this meta‐analysis include: (1) the reduced risk outcomes were consistent across all genetic models; (2) in 95% of the included studies, the genotype data were HWE‐compliant; (3) the aggregate sample sizes of the significant outcomes had statistical power above the set threshold of 75%; (4) the efficiency of outlier treatment was key to generating associative significance and reducing or eliminating heterogeneity; (5) applying the Bonferroni correction reduced the risk of Type I error; (6) the absence of significant covariates in a meta‐regression suggests that the results are consistent across studies, regardless of differences in the tested covariates; and (vii) the core outcomes were robust and showed no evidence of publication bias.

### 4.5 Practical Applications

From a clinical perspective, our findings delineate the influence of *BDNF* polymorphisms on the risk of AUD. Genetic testing of this polymorphism and blood BDNF protein levels in AD patients may provide new approaches for prognostic markers to improve therapeutic strategies for the prediction, prevention, and management of AUD.

## 5 Conclusion

This meta‐analysis, focusing on the four SNPs of the *BDNF* gene (rs6265, rs16917204, rs7103411, and rs11030104) in complete LD, shows a significant association between *BDNF* polymorphisms and AUD. Specifically, our results suggest a protective effect of the homozygous and codominant models of the *BDNF* gene for AUD in the overall analysis and the homozygous and recessive models in the Caucasian subgroup. No association was observed in the Asians population.

Future high‐throughput studies focusing on novel genetic and epigenetic variants of functional relevance, such as exome sequencing, miRNA profiling, and DNA methylation analyses, combined with meta‐analyses of quantitative endophenotypes, could uncover additional molecular susceptibility factors for AUD.

## Author Contributions

**Anorut Jenwitheesuk**: conceptualization, data curation, investigation, validation, writing–original draft, writing–review & editing, funding acquisition. **Noel Pabalan**: conceptualization, data curation, formal analysis, investigation, methodology, software, resources, supervision, visualization, writing–review & editing. **Pairath Tapanadechopone**: supervision. **Hamdi Jarjanazi**: methodology, resources. **Kittipun Arunphalungsanti**: data curation, validation. **Phuntila Tharabenjasin**: conceptualization, writing–original draft, writing–review & editing, investigation, methodology, visualization, software, formal analysis, project administration, data curation, supervision, resources.

## Conflicts of Interest

The authors declare no conflicts of interest.

## Ethics Statement

Not applicable

### Peer Review

The peer review history for this article is available at https://publons.com/publon/10.1002/brb3.70359

## Supporting information



Supporting Information

## Data Availability

The raw data for meta‐analysis in this study are available from the corresponding author upon reasonable request.
